# Spanish Validation of the COMM Scale to Assess the Misuse of Prescription Opioids in Patients with Chronic Noncancer Pain

**DOI:** 10.1007/s11469-022-00803-3

**Published:** 2022-03-21

**Authors:** Ángela Reyes-Pérez, Alicia E. López-Martínez, Rosa Esteve, Carmen Ramírez-Maestre

**Affiliations:** 1 Departamento de PersonalidadEvaluación Y Tratamiento PsicológicoFacultad de Psicología Y Logopedia, Universidad de Málaga, Málaga, Spain; 2 Departamento de PersonalidadEvaluación Y Tratamiento PsicológicoFacultad de Psicología Y Logopedia, Universidad de Málaga, Instituto de Investigaciones Biomédicas de Málaga (IBIMA), Campus de Teatinos, 29071 Málaga, S/N Spain

**Keywords:** Opioid therapy, Opioid misuse, Spanish COMM, Assessment, Psychometric properties

## Abstract

The Current Opioid Misuse Measure (COMM) is a questionnaire used to identify and monitor chronic pain patients on opioid therapy who may be misusing their prescription opioids. The aim of the present study was to adapt the questionnaire for its use in Spanish-speaking populations. A total of 171 individuals (131 women and 40 men) with nononcological chronic pain participated in this cross-sectional study. The most frequent pain diagnoses in the sample were fibromyalgia, herniated disc, and rheumatoid arthritis. Systematic sampling was used. All individuals were interviewed at their clinic between March 2018 and February 2020. The dimensionality of the COMM-SV items was evaluated using an optimal implementation of parallel analysis (PA) and an exploratory factor analysis. Internal consistency, test–retest reliability, and criterion and convergent validity were calculated. The COMM-SV comprises five factors: problematic interpersonal behaviour, searching for more medication than prescribed, medication misuse and self-injurious thoughts, emergency use, and memory and attention problems. It has good reliability and adequate test–retest stability. The results support its criterion and convergent validity. Given the increasing use and abuse of opioids, a valid and reliable instrument is needed in Spanish settings to identify patients with chronic pain who present aberrant behaviour related to the use of these medications. The COMM-SV enables Spanish clinicians to do that.

Opioids are used as a potentially effective treatment for chronic pain (Chou et al., 2009; Volkow & McLellan, 2016). Chronic pain persists or recurs for longer than 3 months and is a multifactorial condition. Thus, biological, psychological, and social factors contribute to the pain syndrome (Treede et al., 2019). Over the last 2 decades, there has been an increase in opioid prescription and consumption worldwide, including in Spain (Ministerio de Sanidad, 2019). This global problem is generating great concern in the social and health systems due to the addictive potential of opioids, their inappropriate use, and the increase in death by opioid overdose in some countries (Acuña, 2019; Guardia, 2018; Santana Pineda et al., 2016).

The initial diagnosis and management of chronic pain is generally the responsibility of primary care physicians, and many of them have reported that they are not adequately trained to recognize and manage patients at high risk of or experiencing prescription drug use disorder (Meltzer et al., 2011; O'Brien et al., 2017). Although opioid therapy is effective, opioids are addictive substances (Manchikanti et al., 2012). Aberrant opioid-related behaviour is generally related to the misuse or abuse of medications (i.e. not following medical prescriptions) but may also include diversion activities (e.g. sharing or selling medications) and drug-seeking behaviours (e.g. seeking pain medicine from multiple providers or visiting emergency department to obtain additional prescriptions) (Turk et al., 2008). Therefore, identifying and monitoring chronic pain patients who may be carrying out such behaviour is key to preventing misuse, abuse, addiction, overdose, and other analgesic-related problems (O'Brien et al., 2017). On the other hand, mood, anxiety, or psychological trauma-related disorders, as well as transdiagnostic variables such as anxiety sensitivity, distress intolerance, pain-related anxiety, and pain catastrophizing, have been postulated as being comorbid with substance abuse and chronic pain (Ditre et al., 2019). Thus, there are recent findings that demonstrate a role of distress intolerance in opioids. Particularly, in the specific context of chronic pain, it has been demonstrated that higher levels of distress intolerance were related to more likely and severity of prescribed opioid misuse, even when controlling for pain intensity and negative affect (McHugh et al., 2016).

Previous reliable and validated assessment tools, such as the Current Opioid Misuse Measure (COMM) (Butler et al., 2007), have been developed to address opioid medication addiction by identifying cases of misuse and organizing preventive measures and specific treatments (Chang & Compton, 2013). The COMM is a self-report instrument to monitor chronic pain patients on opioid therapy and to help identify and manage behaviour related to aberrant medication use (Butler et al., 2007; Chou et al., 2009; Weaver & Schnoll, 2007). 

The COMM was based on the contributions of pain and addiction specialists. They developed 117 items whose conceptual mapping identified six main concepts underlying medication misuse: (1) signs and symptoms of drug misuse, (2) emotional problems/psychiatric issues, (3) poor response to medications, (4) evidence of lying and illicit drug use, (5) inconsistent appointment patterns, and (6) medication misuse/abuse and nonadherence to medication. A total of 40 items were selected from the most relevant ones. The reliability-test–retest showed that 17 items appeared to provide an accurate measure of aberrant behaviour as demonstrated by good internal consistency (Cronbach’s alpha = 0.86) and 1-week test–retest reliability (intraclass correlation coefficient [ICC] = 0.86; 95% CI: 0.77–0.92) (Butler et al., 2007). Recently, Rogers et al. (2020) analysed the factorial structure of the COMM. Their results indicate that the 2-factor structure provided the best solution with two dimensions: (a) problematic drug use and (b) psychiatric problems. Internal consistency was very good (Cronbach’s alphas = 0.96 and 0.90, respectively). Previous research has shown that the COMM can very accurately identify chronic pain patients who have aberrant opioid-related behaviours (Butler et al., 2007). Regarding the validity of the COMM, in the aforementioned study by Rogers et al. (2020), the authors found a significant correlation between both COMM subscales (i.e. aberrant drug-related behaviour and psychiatric problems) and anxiety and depression scores.

As far as we know, and apart from the English version, the COMM has only been validated in China (Zhao et al., 2015) and Portugal (Mendes-Morais et al., 2020). No Spanish study has examined the factor structure of the COMM. Therefore, the main purpose of this study was to analyse the psychometric characteristics of the Spanish version of the COMM (COMM-SV) with three aims: (1) to analyse the factor structure of the COMM-SV, (2) to examine its reliability (internal consistency and test–retest stability), and (3) to examine its validity. Therefore, evidence on the reliability and validity of the COMM was examined based on its internal structure and relation to other variables. As mentioned, previous research has suggested that individuals with anxiety and depression are more prone to develop opioid abuse (Carlson et al., 2016; McHugh et al., 2021; Sullivan et al., 2006). In addition, distress intolerance, considered a dispositional variable, has been shown to be related to opioid misuse in chronic pain patients who have been prescribed with opioids (McHugh et al., 2016, 2020). For all these reasons, we expected to find positive relationships between the misuse of medication as evaluated with the COMM-SV and pain intensity, anxiety, depression, and distress intolerance in individuals with chronic pain receiving long-term opioid treatment. We also expected to find a positive association between the COMM-SV total score and other measures that assess abuse (i.e. the Drug Abuse Screening Test, DAST-10; Skinner, 1982) and the risk of opioid abuse (i.e. the Screener and Opioid Assessment for Patients with Pain-Revised, SOAPP-R; Butler et al., 2013), which would support the concurrent or convergent validity of the COMM-SV. The results of this study will extend empirical evidence on the COMM by providing new data from Spanish samples.

## Methods

### Participants

The study sample was made up of 171 people (131 women and 40 men) with chronic nononcological musculoskeletal pain. The participants were outpatients referred by physicians from primary care health centres and from a pain unit of a general hospital in Spain. Participants were eligible for the study if they met the following conditions: (a) experiencing chronic noncancer pain, (b) prescription for pharmacological treatment with opioids for more than 90 days, (c) being over 18 years old, and (d) sufficient competence in the Spanish language (spoken and written). The exclusion criteria were as follows: (a) chronic oncological pain, (b) musculoskeletal and/or neuropathic lesions that require immediate surgery, and (c) individuals who were being treated for an oncological disease, a degenerative and/or terminal disease, or with a serious mental disorder (involving loss of consciousness, the sense of reality, and the capacity to be self-sufficient).

The patients’ mean age was 60.29 years (*SD* = 16.13; age range = 34–84), and mean pain duration was 16.13 years (*SD* = 12.83; pain duration range = 1–60). The most frequent pain diagnoses among noncancer chronic pain patients were fibromyalgia (*n* = 65), spinal pain (*n* = 54), arthrosis (*n* = 12), and rheumatoid arthritis (*n* = 12). At the time of the study, 81% were married or cohabiting. Regarding their work status, 41% were retired, 19% were active workers, and 11% were unemployed. In total, 56% had completed primary education, 32% had completed high school, and 12% had a university degree.

### Instruments

#### Current Opioid Misuse Measure (COMM)

The COMM (Butler et al., 2007) is a 17-item measure of aberrant medication-related behaviours that can be summed to create a total score. Each item is rated on a 5-point scale from 0 (*never*) to 4 (*very often*). A total score equal to or more than nine (≥ 9) accurately identifies approximately 80% of patients who are at high risk of aberrant medication-related behaviour (i.e. medication misuse, abuse, addiction, and opioid-seeking behaviour). It also has a sensitivity and specificity of 77% and 66%, respectively (Butler et al., 2007; Chou et al., 2009). In addition, the Cronbach’s alpha value (α) was 0.86, and the 1-week test–retest for the total COMM score was excellent (*ICC* = 0.86 [95% CI: 0.77–0.92]) (Butler et al., 2007). The COMM items were identified empirically based on their ability to accurately identify patients who are engaging in aberrant opioid-related behaviours. A forward–backward translation method was used to adapt this scale to the final Spanish version (Sousa & Rojjanasrirat, 2011). Three native Spanish speakers independently translated the material from English to formal Spanish. The translations were compared and discussed to construct the first version of the Spanish COMM. Subsequently, two native English speakers, who were blinded to the original English instrument, independently translated the Spanish translation back into English. This back translation was compared to the original English COMM to assess conceptual and literal similarities.

#### Pain Intensity

Patients were asked to rate their mildest, moderate, and strongest pain during the previous week, as well as their current pain, on a scale ranging from 0 (*not at all*) to 10 (*extremely painful*) in order to obtain a composite pain index. This scale has been validated to measure pain in individuals with chronic pain. Numerical rating scales are commonly used in pain research and are known to provide valid and reliable measures of pain intensity across different populations (Jensen & Karoly, 2011).

#### The Hospital Anxiety and Depression Scale (HADS)

The HADS (Zigmond & Snaith, 1983) is a 14-item self-reporting scale comprising two 7-item Likert subscales, one for anxiety and one for depression. The Spanish version of the scale used in this study has suitable reliability and validity, with good internal consistency for both scales (*α* = 0.86 for anxiety and *α* = 0.86 for depression) (Quintana et al., 2003). In this study, depression and anxiety had Cronbach’s alpha values of 0.84 and 0.81, respectively.

#### The Distress Tolerance Scale (DTS)

The Spanish version of the DTS (Sandin et al., 2017) was used in the current study. The DTS is a 15-item measure that assesses the degree to which a person experiences and endures psychological states of emotional distress. Each item is rated on a 5-point scale ranging from 1 (*strongly disagree*) to 5 (*strongly agree*), and higher scores are indicative of lower tolerance. The confirmatory factor analysis of the Spanish DTS found four lower-order factors of tolerance, appraisal, regulation, and absorption that loaded onto a higher-order general factor. It showed good internal consistency (*α* = 0.86 for the total DTS score and 0.83, 0.89, 0.84, and 0.83 for tolerance, absorption, appraisal, and regulation, respectively) and adequate temporal stability (7-month test–retest) (*r* = 0.70 for the global DTS). In this study, the total scale was used (Cronbach’s alpha value = 0.87).

#### Screener and Opioid Assessment for Patients with Pain-Revised (SOAPP-R)

The Spanish version was used in this study (Butler et al., 2013). This questionnaire was developed to aid physicians in predicting aberrant medication-related behaviour in chronic pain patients (Butler et al., 2008). Each of the 24 SOAPP-R items asks about the past 30 days and is scored on a five-point Likert scale ranging from 0 (never) to 4 (very often). Respondents are considered to be at risk of aberrant behaviour if they meet or exceed a cut-off score of 18. The reliability of the SOAPP-R was found to be highly significant (test–retest reliability = 0.91; *α* = 0.86) (Butler et al., 2009). In this study, the scale had a Cronbach’s alpha value of 0.82.

#### Drug Abuse Screening Test (DAST-10)

The DAST-10 is a short version of the Drug Abuse Screening Test (Skinner, 1982). It is a 10-item self-report questionnaire that assesses problems related to drug abuse during the past year with two response options for each item (YES/NO). The total score of the measure is obtained by summing all items. The Spanish version of the scale used in this study (Pérez-Gálvez et al., 2010) has been shown to be a valid and reliable instrument in the detection of drug abuse in adult populations. The Spanish version of the DAST-10 showed high internal consistency (α = 0.89) (Pérez-Gálvez et al., 2010). This scale was only completed by a subsample of 44 participants (Cronbach’s alpha = 0.70).

### Procedure

The study was conducted in accordance with the Declaration of Helsinki and received ethical clearance by the Institutional Ethics Review Board (ERC UMA) and the Regional Hospital Ethics Committee.

At the end of their medical visit, all participants who fulfilled the eligibility criteria were informed by their doctor of the study aims, and their participation was requested. The inclusion and exclusion criteria were taken into account by the physicians, according to the information contained in the patients’ medical records before inviting the patients to take part in the study. The participants who accepted were contacted by telephone to make an appointment for the assessment, which was conducted by a trained psychologist (i.e. Master’s degree in clinical psychology) at their clinic (i.e. primary care health centre or the pain unit of a general hospital). All participants were guaranteed confidentiality, and written informed consent was obtained in accordance with the Declaration of Helsinki.

Each participant had a semi-structured interview with trained psychologists to obtain demographic, social, and medical history data (Table 1). Subsequently, the participants completed a battery of questionnaires. All individuals were interviewed at their clinic.

**Table 1:** Demographic and clinical characteristics of the participants

	*M (SD)*	*N (%)*
Age (years)	60.29 (16.13)	
Sex		
Man		40 (23)
Woman		131 (77)
Marital status		
Single		9 (5)
Married/cohabiting		130 (81)
Separated/divorced		19 (11)
Widowed		13 (8)
Education		
Primary school		98 (56)
High school		54 (32)
University degree		19 (12)
Current occupation		
Active worker		31 (19)
Housework		49 (29)
Retired		69 (41)
Unemployed		22 (11)
Pain diagnosis		
Arthrosis		12 (6)
Fibromyalgia		65 (38)
Rheumatoid arthritis		12 (7)
Spinal pain		54 (31)
Others		28 (18)
Length of pain (years)	16.13 (12.83)	

A total of 27 participants were randomly invited to attend a second interview 1 month later by phone to complete the COMM-SV questionnaire again. The data were collected between March 2018 and February 2020. A subsample of 43 participants who had been assessed 18 months before were contacted by telephone in December 2020, due to the restrictions imposed by the COVID-19 pandemic. These individuals were asked for information about medication and pain intensity and were administered the DAST-10.

### Data Analysis

The data were analysed using the SPSS statistical program (Windows version 25.0). Parallel analysis (PA) was conducted using the FACTOR statistical program (version 10.10.02) (Lorenzo-Seva & Ferrando, 2013). The mediation model was tested using the SPSS macro PROCESS (Hayes, 2014).

We calculated the descriptive statistics and distributional properties of the items of the COMM-SV. Raw item-rest correlations were investigated to identify items with relatively smaller multiple correlations with other items for possible exclusion in further analyses.

The number of dimensions was assessed using indices based on PA. Thus, the dimensionality of the COMM-SV items was evaluated using an optimal implementation of PA (Timmerman & Lorenzo-Seva, 2011) using exploratory robust maximum likelihood (RML). An exploratory factor analysis (EFA)—principal axis method—with promax (oblique) factor rotation was conducted to allow for correlations between factors. Goodness-of-fit was evaluated using the following indices: root mean square error of approximation (RMSEA), the comparative fit index (CFI), the non-normed fit index (NNFI), and the standardized root mean square residual (SRMR). Model fit was defined according to the following criteria (Hu & Bentler, 1999): an RMSEA value equal to or less than 0.06 indicates a good fit, CFI and TLI values close to or more than 0.95 indicate an acceptable fit, and an SRMR value close to or less than 0.05 indicates an good fit.

Internal consistency was calculated using Cronbach’s alpha. Test–retest stability estimates were based on data from a subsample (*n* = 27) of the participating patients who completed the first and the second administration (1 month later). The ICC for test–retest reliability was calculated using baseline and 1-month post-assessment scores. Test–retest reliability is considered to be acceptable with scores equal to or greater than 0.65 (Hernández et al., 2016).

Criterion validity was assessed by calculating Pearson correlations between the COMM-SV and scores on pain intensity, depression and anxiety symptoms, and distress tolerance. We also assessed correlations between the SOAPP and the COMM-SV total scores. We followed the guidelines provided by Evers et al. (2013) for interpreting correlations, wherein validity values can be considered inadequate (*r* < 0.20), adequate (0.20 *r* < 0.35), good (0.35 *r* < 0.50), or excellent (*r* > 0.50).

Criterion validity was also assessed using a four-step hierarchical multiple regression. The predictor variables were pain intensity, distress tolerance, anxiety symptoms, and depression symptoms. To control for potential confounders, age and sex (coded as man = 0 and woman = 1) were entered in the first block. In order to estimate the contribution of pain intensity, distress tolerance, and anxiety and depression according to their relevance in the prediction of COMM-SV scores, we entered these variables in the second, third, and fourth block, respectively. Pain intensity was introduced first to control for its effect. Distress tolerance was entered in the second block because it is considered to be a dispositional variable. Anxiety and depression were introduced in the third block. The mediation model was used to investigate the indirect effects of distress tolerance on COMM-SV scores through anxiety symptoms. Direct and indirect effects were estimated using Preacher and Hayes’ techniques with 5000 bootstrap samples (Preacher & Hayes, 2004). Mediation effects were further evaluated using bias-corrected bootstrap 95% confidence intervals (CI). These effects were considered statistically significant if the confidence intervals did not contain zero. Finally, the DAST-10, which provides an index of substance abuse problems, was used to calculate convergent or concurrent validity of the COMM-SV.

## Results

### Descriptive Statistics

We calculated the descriptive statistics of each item of the COMM-SV. Table 2 shows the means, standard deviations, item-test correlations, and reliability of the scale if the item is removed. Item means ranged from 2.46 to 0.18 (items 1 and 9, respectively).

**Table 2:** Descriptive statistics, factor loading after oblique (promax) rotation, and reliability of COMM-SV items

Descriptive statistics Factor loadings									
Items	*M*	*SD*	1	2	3	4	5	*h*^2^	*α*-i
1	2.4588	1.24085	− .123	.024	.134	− .026	.428	.278	.791
2	1.2000	1.21431	.157	− .006	.126	− .127	.439	.415	.779
3	.5882	.98262	.051	.483	.061	.333	.016	.453	.776
4	.9765	1.10380	.053	.016	.806	− .055	− .116	.442	.777
5	.6176	.99747	.115	− .126	.342	− .034	.230	.366	.782
6	1.3882	1.29719	− .071	.214	.339	.123	.184	.450	.776
7	1.5647	1.10884	.939	− .011	.028	− .005	− .074	.506	.772
8	.7765	.95925	.627	.012	.003	− .028	.053	.420	.779
9	.1824	.61224	− .068	.803	.040	− .062	.019	.365	.784
10	.8529	1.14954	.077	− .017	− .204	.002	.673	.272	.790
11	.5882	1.04677	− .058	.012	.007	.059	.691	.402	.780
12	.4118	.78889	− .031	− .033	− .026	.921	.027	.271	.788
13	1.4059	1.11736	.773	.022	.038	.073	.017	.540	.769
14	.8765	1.09977	− .035	− .023	.949	.021	− .020	.539	.769
15	.1765	.55845	.069	.985	− .099	− .104	− .035	.388	.784
16	.5176	.89193	.064	− .015	.169	− .095	.037	.136	.796
17	.3765	.73744	.051	− .051	− .043	.895	− .047	.250	.789

h^2^ = communalities; *α* -i = reliability of the scale if the item is removed

### Parallel Analysis

The PA using exploratory RULS indicated a 5-factor structure. According to the goodness of fit indexes, the model showed a very good fit: χ^2^ was nonsignificant (*χ*^2^ = 71.79, *p* = 0.16, *RMSEA* = 0.03, *SRMR* = 0.04, *CFI* = 0.99, and NNFI = 0.98).

### Factor Structure

The Kaiser–Meyer–Olkin (KMO) index was 0.70, indicating that the EFA was adequate for this sample.

The EFA yielded five factors with eigenvalues > 1. This solution accounted for 53.4% of the variance (with factors 1, 2, 3, 4, and 5 explaining 22.41, 9.87, 8.46, 6.49, and 6.21 of the variance, respectively) and with eigenvalues of 4.20, 1.97, 1.76, 1.60, 1.38, and 1.07 for factors 1, 2, 3, 4, and 5, respectively. All loadings were greater than 0.30 except for item 16 (factor loading 0.17), and communalities were between 0.27 (item 12) and 0.54 (item 13), except for item 16 (communality 0.14). Factorial correlations values were between 0.15 (factor 3 and factor 4) and 0.37 (factor 1 and factor 3). Factor 1 consisted of 3 items (7, 8, 13) on problematic interpersonal behaviour; factor 2 comprised 3 items (3, 9, 15) on searching for more medication than prescribed; factor 3 consisted of 5 items (4, 5, 6, 14, 16) on medication misuse and self-injurious thoughts; factor 4 comprised 2 items (12, 17) on emergency use; and factor 5 consisted of 4 items (1, 2, 10, 11) on memory and attention problems. Table 2 shows the descriptive statistics for the items, the EFA results, and reliability of the scale if the item is removed. Item means ranged from 2.46 to 0.18 (items 1 and 15, respectively).

### Internal Consistency

Cronbach’s alpha was calculated for the COMM-SV and its five subscales. The total score of the questionnaire showed suitable internal consistency (*α* = 0.80). The internal consistency for the factors ranged from *α* = 0.64 (factor 5) to *α* = 0.89 (factor 4).

### Test–Retest Reliability

The ICC for the test–retest reliability of the COMM-SV total score (total and factors scores) was calculated using baseline and 1-month post-assessment scores. Measurements were repeated twice for each participant. ICC test–retest reliability was high (0.97; 95% CI: 0.94–0.99).

### Criterion Validity

Table 2 shows the descriptive statistics and correlations between all the variables measured. As shown in Table 3, a significant high positive correlation was found between the COMM-SV scores and the SOAPP-R total score (i.e., the risk of opioid abuse) and anxiety symptoms, a moderate positive association between the COMM-SV scores and the DTS total score and depression symptoms, and a significant low positive association between the COMM-SV scores and pain intensity. Thus, the associations between variables were as expected, thereby supporting the criterion validity of the COMM-SV.

**Table 3:** Means, standard deviations, and correlations between the main study variables

Variables	Mean (*SD*)	1	2	3	4	5	6
COMM-SV	14.95 (8.29)	1					
Pain intensity	7.39 (1.34)	.17*	1				
Distress tolerance (total score)	49.37 (14.32)	.44**	.13	1			
Anxiety symptoms	19.92 (5.64)	.53**	.27**	.62**	1		
Depression symptoms	16.50 (4.76)	.44**	.27**	.57**	.60**	1	
SOAPP-R	30.27 (11.86)	.58**	.22**	.56**	.66**	.54**	1

*SOAPP-R* Screener and Opioid Assessment for Patients with Pain-Revised

^**^ *p* < .01; * *p* < .05

Table 4 shows the results of the regression analysis for the prediction of opioid misuse (measured with the COMM-SV). The results of the assumptions testing showed that the values of the variance inflation factors (1.08–1.85) in the regression analyses were less than the standard cut-off of 10 (Hair et al., 1995), indicating an absence of multicollinearity between the predictor variables. Durbin-Watson values ranged between 1.5 and 2.5 for the criterion variables (opioid misuse: 1.84). After controlling for demographic variables (age and sex) in step two, pain intensity (*β* = 0.18, *p* = 0.022) significantly contributed to the prediction of opioid misuse. However, in step 3, when distress tolerance was included in the equation, this new variable alone (*β* = 0.45, *p* < 0.001), but not pain intensity (*β* = 0.13, *p* = 0.071), significantly contributed to the prediction of opioid misuse (∆*R*^2^ = 0.20). Finally, in step 4, anxiety symptoms alone (*β* = 0.40, *p* < 0.001), but not depression symptoms (*β* = 0.14, *p* = 0.096), made an additional significant contribution to the prediction of the criterion variable (∆R^2^ = 0.09). In this study, distress tolerance and anxiety symptoms explained 32% of opioid misuse variance.

**Table 4:** Results of multiple regression analysis predicting opioid misuse (COMM-SV)

Criterion variable	Predictive variables	*β*	*R*^*2*^	*F*
COMM-SV				25.39**
	*Model 1*		.03	
	Pain intensity	.18*		
	*Model 2*		.23	
	Pain intensity	.13		
	Distress intolerance	.45**		
	*Model 3*		.32	
	Pain intensity	.05		
	Distress intolerance	.21*		
	Anxiety symptoms	.40**		

^*^ *p* < .05; ** *p* < .01

**Fig. 1:**
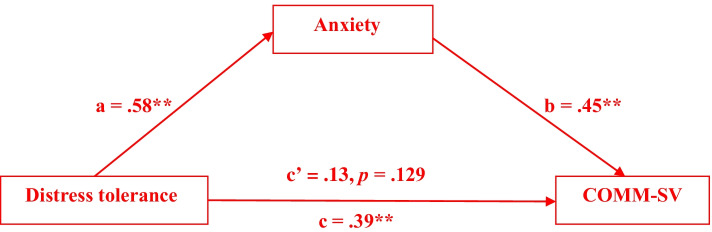
Completely standardized indirect effect of distress tolerance on COMM-SV. ** *p* < .001

Figure 1 shows the results of the mediation analysis as well as the path coefficients tested in the model. It was found that distress tolerance was significantly associated with opioid misuse as measured with the COMM-SV (path c; *b* = 0.25, *SE* = 0.05, *p* < 0.001). Distress tolerance was also associated with anxiety (path a; *b* = 0.25, *SE* = 0.03, *p* < 0.001), and anxiety was shown to be associated with COMM-SV (path b; *b* = 0.68, *SE* = 0.13, *p* < 0.001). Thus, anxiety symptoms had a significant mediating effect between distress tolerance and the COMM-SV. Given that the direct effect between distress tolerance and opioid misuse was not significant when the indirect effect of anxiety was included (path c’; *b* = 0.08, *SE* = 0.06; 95% CI [− 0.03, 0.19]), it can be concluded that anxiety completely mediated the relationship between distress tolerance and opioid misuse.

### Concurrent Validity

Pearson’s bivariate correlation between the COMM and DAST-10 total scores yielded a value of *r* = 0.48 (*p* < 0.01), indicating a moderate positive association between both measures.

## Discussion

The main objective of the present study was to analyse the psychometric characteristics of the Spanish version of the COMM, which is a questionnaire that identifies chronic noncancer pain patients who misuse opioid prescriptions. This instrument detects aberrant behaviour related to prescribed opioids and serves as a tool for monitoring medical treatment. To our knowledge, the present study is the first to analyse the structural validity of the COMM using data from a sample of Spanish patients with chronic pain.

As indicated by the factor analysis of the COMM-SV, the 17 items are grouped into five factors. This factor structure is different from the ones found in previous studies. According to Rogers et al. (2020), the original English version of the COMM (Butler et al., 2007) has a 2-factor structure, the Chinese version (Zhao et al., 2015) has a 4-factor structure, and the Portuguese version (Mendes-Morais et al., 2020) has a 6-factor structure. All these studies include factors related to emotional, cognitive, and interpersonal problems and factors related to medication misuse (including searching for more medication than prescribed). In addition, in all versions of the COMM, the total score must be calculated—regardless of the number of factors—to identify patients who are engaging in aberrant opioid-related behaviours. Further, regardless of the number of factors obtained in the different countries, all COMM versions show good internal consistency (ranging from 0.86 for the original version to 0.78 for the Portuguese version).

Regarding convergent validity, an expected significant association was found between the total scores on the COMM-SV total score and the DAST-10 scale (Sandin et al., 2017), which is a tool for screening drug abuse. The results showed a positive association between the scores of both instruments. Therefore, the COMM-SV showed good convergent validity.

We assessed the criterion validity of the COMM-SV by investigating correlations between the risk of misuse as measured with the SOAPP (Butler et al., 2013) and the COMM-SV total scores. As expected, there was a correlation between a high risk of misuse and high scores on the COMM-SV. This result supports the validity of the COMM-SV. We also tested a number of hypotheses regarding the relationship between pain intensity, depression and anxiety symptoms, distress tolerance, and the total score of the COMM-SV. The results confirmed these hypotheses. Firstly, a significant positive association was found between higher levels of pain intensity, depression, anxiety, distress tolerance, and higher scores on the COMM-SV. These findings are consistent with previous studies that have shown that opioid abuse is more prone to develop in individuals with chronic pain who are receiving long-term opioid treatment and have anxiety, depression, and/or stress intolerance (Carlson et al., 2016; McHugh et al., 2016; Sullivan et al., 2006). Secondly, the results of the regression analysis show that both distress tolerance and anxiety symptoms are associated with opioid misuse as measured with the COMM-SV. Finally, the mediation hypothesis tested showed that the relationship between distress tolerance and opioid misuse was mediated by anxiety symptoms. The results support this hypothesis because anxiety mediated 100% of the relationship between distress tolerance and opioid misuse. These findings are in line with previous studies that have suggested that distress tolerance is related to higher pain-related anxiety, thereby affecting substance use processes in individuals with opioid use disorder (Langdon et al., 2019; McHugh et al., 2016). Langdon et al. (2019) found that people with high levels of distress tolerance are more prone to therapeutic opioid abuse. Thus, it seems that these individuals are more prone to therapeutic opioid abuse and that they could use this medication trying to relieve anxiety. According to all the results, psychological variables, such as anxiety, play a key role in opioid misuse/abuse, which is in line with previous findings (for a recent review, see McHugh et al. (2021)). Consequently, our results seem to suggest that clinicians should screen patients’ psychological variables before beginning opioid treatment. The accurate and early psychological evaluation of patients could help to decide whether opioid treatment is indicated and also help to determine the level of monitoring needed based on the severity of the patients’ psychological symptoms.

This study has some limitations that should be considered. Firstly, the size of the clinical sample was relatively small. Secondly, women were overrepresented in the sample, which may have biased the findings. Future research should replicate these results using a larger sample with more male participants. Thirdly, only self-report measures were used, and so the results may be biased due to shared method variance. Fourthly, the cross-sectional and correlational design employed cannot be used to make causal statements.

Despite these limitations, this study represents the first attempt to validate the COMM-SV. The findings suggest that the Spanish version of the COMM provides a reliable measure that can help clinicians evaluate and identify patients with aberrant behaviour related to the use of opioid medication. Given the growing concern in Spain on the use and abuse of opioid analgesics (Santana Pineda et al., 2016), it is essential to have a reliable and valid instrument, such as the COMM-SV, to detect misuse.
